# The Community of Hymenoptera Parasitizing Necrophagous Diptera in an Urban Biotope

**DOI:** 10.1673/031.013.3201

**Published:** 2013-04-18

**Authors:** Christine Frederickx, Jessica Dekeirsschieter, François J. Verheggen, Eric Haubruge

**Affiliations:** Department of Functional and Evolutionary Entomology, Gembloux Agro-Bio Tech, University of Liege, Passage des Déportés 2, 5030 Gembloux, Belgium

**Keywords:** *Alysia manducator*, carrion ecology, forensic entomology, *Nasonia vitripennis*, *Tachinaephagus zealandicus*, temperate area

## Abstract

Most reports published in the field of forensic entomology are focused on Diptera and neglect the Hymenoptera community. However, Hymenoptera are part of the entomofaunal colonization of a dead body. The use of Hymenoptera parasitoids in forensic entomology can be relevant to evaluate the time of death. Hymenoptera parasitoids of the larvae and pupae of flies may play an important role in the estimation of the post-mortem period because their time of attack is often restricted to a small, well-defined window of time in the development of the host insect. However, these parasitoids can interfere with the developmental times of colonizing Diptera, and therefore a better understanding of their ecology is needed. The work reported here monitored the presence of adult Hymenoptera parasitoids on decaying pig carcasses in an urban biotope during the summer season (from May to September). Six families and six species of parasitoids were recorded in the field: *Aspilota fuscicornis* Haliday (Braconidae), *Alysia manducator* Panzer, *Nasonia vitripennis* Walker (Pteromalidae), *Tachinaephagus zealandicus* Ashmead (Encyrtidae), *Trichopria* sp. (Diapriidae), and *Figites* sp. (Figitidae). In the laboratory, five species emerged from pupae collected in the field: *Trichopria* sp., *Figites* sp., *A. manducator, N. vitripennis*, and *T. zealandicus*. These five species colonize a broad spectrum of Diptera hosts, including those species associated with decomposing carcasses, namely those from the families Calliphoridae, Muscidae, Fanniidae, and Sarcophagidae.

## Introduction

As soon as an animal dies, the carcass becomes a food source for many organisms (Grassberger and Frank 2004; Carter et al. 2007). In temperate natural biotopes, the most specialized organisms inhabiting the “cadaver-ecosystem” are insects (Amendt et al. 2004). Necrophagous insects, mainly Diptera and Coleoptera, are attracted to the cadaver, which is then colonized in a relatively predictable sequence called the entomofaunal succession or insect succession (Megnin 1894; Putman 1983; Schoenly and Reid 1987; Marchenko 1988, 2001; Benecke 2004). Study of these insects in a medico-legal context is a component of forensic entomology (Hall 1990; Amendt et al. 2004). Many forensic entomological studies have been conducted using pig carcasses as surrogate human models due to physiological, ethical, legal, and economic reasons (Rodriguez and Bass 1983; Catts and Goff 1992; Anderson and VanLaerhoven 1996; Hart and Whitaker 2005), but few of the studies conducted on pig carcasses have taken place in Europe (Garcia-Rojo 2004; Grassberger and Frank 2004; Wyss and Cherix 2006; Matuszewski et al. 2008; Dekeirsschieter et al. 2011; Matuszewski et al. 2011).

Many published reports are focused on the Diptera community, but very few look at the parasite community (Davies 1999; Amendt et al. 2000; Campobasso et al. 2001; Grassberger and Frank 2003; Schroeder et al. 2003; Amendt et al. 2004; Wang et al. 2008). Predators and parasites are generally considered to be the second most significant group of carrion-frequenting taxa (Goff 2010, 2011). Among these, a special group of parasites, called parasitoids, attack several necrophagous taxa. A parasitoid larvae or pupae feed exclusively on other arthropods, mainly insects, resulting in the death of the host (Eggleton and Gaston 1990; Amendt et al. 2010). They represent an extremely diverse group, though mainly belonging to Hymenoptera. In Europe, 83 parasitoids species, which attack the larval and pupal stages of synanthropic Diptera, are listed (Fabritius and Klunker 1991). The use of Hymenoptera parasitoids in forensic entomology can be useful to evaluate the time of death (Amendt et al. 2000; Grassberger and Frank 2003; Amendt et al. 2010). The pupal parasitoids of blowflies may play an important role in the estimation of the post-mortem period, because their time of attack is often restricted to a small, welldefined window of time in the development of the host insect (Anderson and Cervenka 2002). This specialized group might also lead to significant problems for forensic entomologists. For example, changes in developmental times for *Lucilia sericata* L. have been observed after attack by the parasitoid *Alysia manducator* Panzer, the result being premature pupation (Holdaway and Evans 1930). Families of Hymenoptera parasitoids of forensic importance include Braconidae, Pteromalidae, and Ichneumonidae (Amendt et al. 2000; Disney and Munk 2004; Turchetto and Vanin 2004). Among them, *Nasonia vitripennis* Walker (Pteromalidae) and *Alysia manducator* Panzer (Braconidae) are the most common parasitoids found on cadavers (Grassberger and Frank 2003; Grassberger and Frank 2004; Turchetto and Vanin 2004).

So far, little information is available on the Hymenoptera post-mortem community in temperate biogeoclimatic countries (Woodcock et al. 2002; Wyss and Cherix 2006; Lefebvre and Gaudry 2009). This paper identifies the Hymenoptera parasitoid community that was identified on large carcasses in a temperate urban biotope during summer.

## Materials and Methods

### Field site and study periods

This study was conducted during summer 2010 (4 May – 30 September) in an abandoned garden at an urban site located in Belgium. The garden consisted of hazel trees (*Corylus avellana* L.), spruce (*Picea* spp.), and ash trees (*Fraxinus excelsior* L.). The shrub layer was absent. The soil vegetation was scattered, and the herb layer was mainly constituted of nettles (*Urtica dioica* L.) and ivies (*Hedera helix* L.). Regarding the moss layer, there were some sparse areas of *Polytrichum* sp.

The ambient air temperature and humidity were automatically measured once an hour using a data logger (HOBO RH/Temp 8K©; Onset Computer Corporation, www.onsetcomp.com) placed on the lateral side of each cage at a height of 75 cm. The daily mean temperature was calculated on the basis of ambient air temperature recorded at time intervals of 24 hr.

### Animal model

Each month, two male piglets, *Sus domesticus* L. (Artiodactyla: Suidae) (5 kg), were killed by penetrative captive bolt (fractured skull) and placed at the experimental sites within two hours. Piglets were provided by the experimental farm of the Veterinary Medicine Faculty of the University of Liege, Belgium (ethical authorization number: FUSAGx-08-07). Immediately after euthanasia, the pig carcasses were packed in double plastic bags to avoid any insect colonization, before being placed at the experimental site.

**Figure 1.**
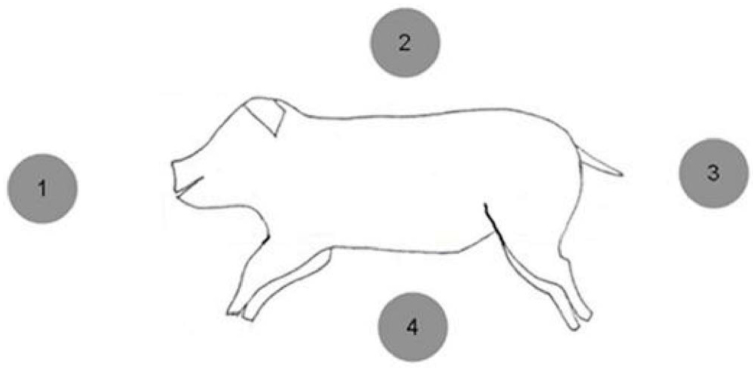
Position of yellow traps around each pig carcass. High quality figures are available online.

Each pig carcass was placed 30 m from each other in a plastic box (50 cm × 95 cm × 50 cm) filled with 30 cm of soil from the site in order to facilitate the collection of pupae samples. This box was placed in a metal mesh cage (180 cm × 90 cm × 90 cm) to avoid scavenging by vertebrate carnivores. Dates of exposure of the piglets were 4 May, 2 June; 30 June; 11 August, and 13 September.

### Insect collection and identification

In order to quantify insect colonization on pig carcasses, four yellow traps (plastic containers of 9 cm height and 27 cm diameter) filled with soapy water were placed around each carcass. The distribution of the yellow traps on the ground was as follows: one near the head, one near the dorsal face, one near the anus, and one near the ventral face (Figure 1). The insect traps were removed every week and the collected specimens were conserved in 80% *norvanol D* (ethanol denatured with ether). Only adult stages were included in the counting of collected insects during this study.

**Figure 2.**
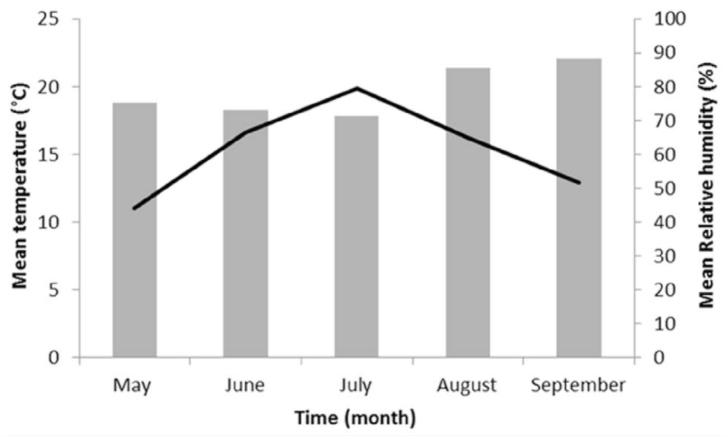
Average monthly ambient temperatures (lines) and average monthly relative humidity (bars). High quality figures are available online.

At the end of each month, pupae present in the soil under the pig carcasses were collected before being transported to the laboratory for rearing. Rearing was conducted under environmentally controlled conditions of 23 ± 1° C with a daylight regime of 16:8 L: D and 70% RH. Pupae were stored together in plastic containers until either an adult fly or parasitoids emerged. Emerged specimens were killed in 80% *norvanol D*. Pupae from which no adult flies or parasitoids had emerged after eight weeks were dissected for evidence.

Emerged Diptera were identified by their species. However, parasitoid hosts were not identified. Typically, the puparium were very similar in general appearance, being coarctate and light-brown to dark-brown in color, which made identification difficult (Sukontason et al. 2007). Moreover, only one pupal identification key exists. This key identified seven fly species of forensic importance in Thailand, such as *Chrysomya* spp. (Sukontason et al. 2007). Hymenoptera were identified by family, or to genera when it was possible. Moreover, the subfamilies of Alysiinae, Pteromalinae, Encyrtinae, Figitinae, and Diapriinae were mounted on insect pins and identified by species. Hymenoptera specimens were determining using different identification keys (Fischer 1971, 1972; Nixon 1980; Fergusson 1986; Delvare and Aberlenc 1989; Goulet and Huber 1993).

## Results

### Environmental parameters

The mean atmospheric temperatures measured during the decompositional process were 11.0° C for May, 16.6 °C for June, 19.9° C for July, 16.2° C for August, and 12.9° C for September (Figure 2). The mean relative humidity was 75.1% for May, 73.2% for June, 71.3% for July, 85.6% for August, and 88.3 % for September (Figure 2).

### Hymenoptera specimens collected in the field

The Hymenoptera superfamilies identified on pig carcasses were Ichneumonidea (one family), Chalcidoidea (two families), Cynipoidea (two families), and Proctotrupoidea (one family) (Table 1). Six families were identified: were identified: Braconidae, Pteromalidae, Encyrtidae, Figitidae, Eucoilidae, and Diapriidae. The richness of variety in seasonal families was shown to be three families in May, five families in June, six families in July, and five families both in August and September.

**Table 1.**
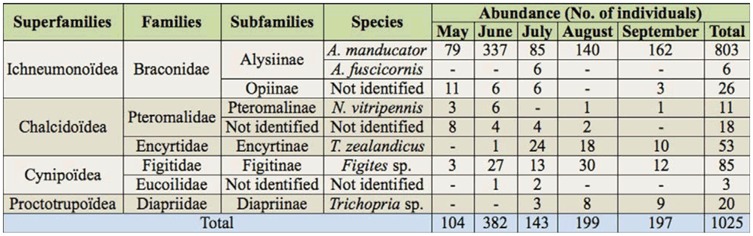
List of Hymenoptera collected in yellow traps.

Six species of Hymenoptera were identified during the sampling period, and subfamilies of Alysiinae included species of *Alysia manducator* Panzer (803 specimens) and *Aspilota fuscicornis* Haliday (Braconidae) (six specimens). *N. vitripennis* (11 specimens), *Tachinaephagus zealandicus* Ashmead (Encyrtidae) (53 specimens), *Trichopria* sp. (Diapriidae) (20 specimens), and *Figites* sp. (Figitidae) (85 specimens) were also identified. *Figites* sp., *Trichopria* sp., and *A. manducator* were collected all through the summer season. *T. zealandicus* was collected from June to September, however, *A. fuscicornis* was collected only during July.

Of the total individuals (Table 1), 10.15% were collected in May, 37.27% in June, 13.95% in July, 19.41% in August, and 19.22% in September. *A. manducator* was the most abundant species overall (78.34%), followed by *Figites* sp. (8.29%) and *T. zealandicus* (5.17%). The remaining families consisted of fewer than 30 collected individuals. In May, *Figites* sp. and *N. vitripennis* were the most abundant species. During June, the predominant species was *Figites* sp., followed by *N. vitripennis*. The most predominant species in July was *T. zealandicus*, followed by *Figites* sp. and *A. fuscicornis*. In August and September, *Figites* sp. was most abundant, followed by *T. zealandicus* and *Trichopria* sp.

### Hymenoptera reared from fly pupae

In total, 13,310 Diptera pupae were collected from the soil under carcasses throughout the study (Table 2). Of these, 47.96% were parasitized by Hymenoptera parasitoids, which yielded 6,833 successfully emerged parasitoid specimens. The percentage of parasitism during the summer season was 3.48% in May, 8.99% in June, 8.88% in July, 49.79% in August, and 90.10% in September.

**Table 2.**
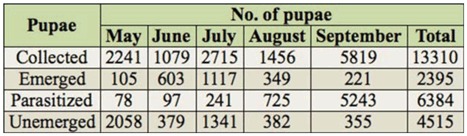
Number of pupae collected, emerged, parasitized, and not emerged in laboratory.

**Table 3.**
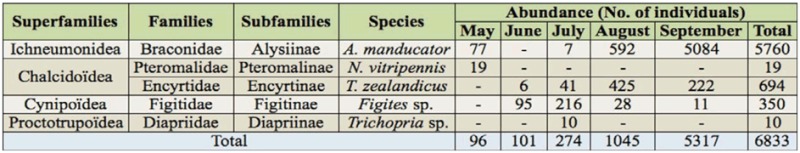
List of Hymenoptera reared from fly pupae.

**Table 4.**
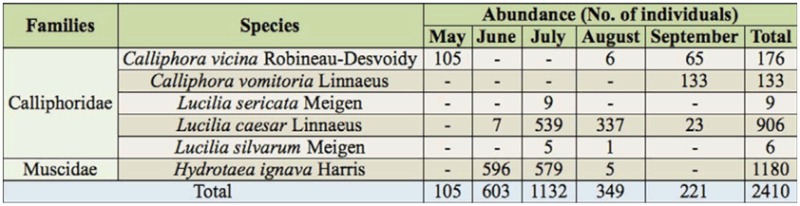
Diptera species emerged in laboratory.

Five parasitoid species emerged from pupal samples collected in the field (Table 3). The number of *A. manducator* collected increased dramatically in September. *N. vitripennis* and *Trichopria* sp. were collected only in May and July. *T. zealandicus* and *Figites* sp. were collected from June to September. *A. manducator* was the predominant species (84.30%), followed by *T. zealandicus* (10.16%) and *Figites* sp. (5.12%). Comparatively, parasitism of fly pupae by *N. vitripennis* and *Trichopria* sp. was rare, with both species contributing less than 0.3% of the total parasitism recorded.

Of the Diptera specimens that emerged in the laboratory, two families were identified in association with carcasses: Calliphoridae with five species, and Muscidae with one species (Table 4). All of these species have previously been reported as carrion breeding flies in Europe (Wyss and Cherix 2006; Lefebvre and Gaudry 2009).

## Discussion

The objective of this study was to document Hymenoptera, and more precisely Hymenoptera parasitoids of necrophagous Diptera, in an urban site. At this site, the Hymenoptera community was represented by six families and was found to change over time. The make-up of the Hymenoptera community differed between months. The Hymenoptera diversity was highest in July, followed by June, August, September, and finally May. However, the lowest abundance of Hymenoptera occurred in May, with approximately three times fewer specimens trapped than in June. June had the highest level of caught insects, followed by August, September, and July. Our breeding in the laboratory showed the highest rate of parasitism was observed in September. All previous reports from Europe have been anecdotal, limited to family level or carrion fauna lists (Woodcock et al. 2002; Wyss and Cherix 2006; Lefebvre and Gaudry 2009).

This study identified five species of parasitoids that visited decomposing remains in search of host carrion flies: *A. manducator, N. vitripennis, Figites* sp., *Trichopria* sp., and *T. zealandicus*. These five species colonized a broad spectrum of Diptera hosts associated with decomposing carcasses, including Calliphoridae, Muscidae, Fanniidae, and Sarcophagidae (Laing 1937; Whiting 1967; Rueda and Axtell 1985; Blanchot 1992; Goulet and Huber 1993; Ferreira De Almeida et al. 2002; Voss et al. 2010).

In the field and laboratory, the most abundant parasitoid species, approximately 78% of the species caught and 84% of the species that emerged, was *A. manducator*. 5,760 pupae were parasitized by *A. manducator* in the field. These females are attracted to decomposing meat (Laing 1937; Blanchot 1992; Reznik et al. 1992). On the carrion, an *A. manducator* female walks over the surface, stabbing frequently with her ovipositor until a host larva is encountered (Laing 1937; Reznik et al. 1992). Hosts are discovered by contact, and female parasites prefer larger size larvae (Graham-Smith 1919; Blanchot 1992). *A. manducator* is an endoparasitoid, and it lays one egg per host (Reznik et al. 1992; Goulet and Huber 1993). This species is present worldwide, but much more prevalent in temperate regions (Goulet and Huber 1993), and has been collected in an urban site in central Europe in May (Grassberger and Frank 2004). In the British Isles, *A. manducator* is the most common of the parasitic Hymenoptera likely to be seen in carrion (Smith 1986). The seasonal activity of *A. manducator* reported in the present study is in agreement with previous observations in the Paris region, where it was active from May to October (Blanchot 1992). In the present study, the peak of it is presence in yellow traps was observed during June (337 specimens).

Three subfamilies are recognized in Figitidae: Anacharitinae, Aspiceratinae, and Figitinae (Goulet and Huber 1993). Aspiceratinae and Figitinae are solitary endoparasitoids of Diptera pupae and early stage larvae, respectively, but the parasitoid emerges from the puparium (Fergusson 1986; Goulet and Huber 1993). Payne and Mason (1971) identified two genera of Figitinae, *Figites* and *Neralsia*, which were collected from pig carcasses. Exposed larvae were generally parasitized; however, *Figites* would also enter the carcass in search of prey. *Neralsia* were only observed parasitizing exposed larvae (Payne and Mason 1971). Six percent of the total caught specimens belonged to the Figitinae subfamily. In the laboratory, 350 pupae were parasitized by one single species *of Figites* sp. These small parasitoids were attracted to carrion where *Lucilia* spp. and *Sarcophaga* spp. larvae were the prevalent species (Payne and Mason 1971). The parasitism of *Figites* sp. in the field corresponded to the time period when *Lucilia* spp. was predominant (June to September) (Payne and Mason 1971).

Encyrtidae is one of the most important chalcidoid families for biological control (Goulet and Huber 1993; Voss et al. 2010). Species are gregarious endoparasitoids of eggs, third instar larvae, and postfeeding or prepupae of several forensically important Diptera (Fanniidae, Muscidae, Calliphoridae) (Goulet and Huber 1993; Ferreira De Almeida et al. 2002; Voss et al. 2010). In 2003, the presence of *T. zealandicus* was detected in northern Italy during September (Turchetto et al. 2003; Turchetto and Vanin 2004). This species, probably native to Australia and New Zealand, has been introduced into various parts of the world in attempts to control pest species of synanthropic Diptera, but no records are available for Europe or the northern regions (Turchetto and Vanin 2010). Following Italy, this is the second recording of *T. zealandicus* in the Palearctic Region (Turchetto et al. 2003).

The family of Diapriidae includes four subfamilies: Belytinae, Ismarinae, Ambositrinae, and Diapriinae (Goulet and Huber 1993). Only Diapriinae contains parasitoid species of necrophagous Diptera (Goulet and Huber 1993). In the field, *Trichopria* sp. made-up approximately 2% of the total specimens caught. In the laboratory, one species of *Trichopria* sp. parasitized only five pupae in July. Moreover, only three specimens were collected in July. These small black insects are endoparasitoids of the immature stages of Diptera (Payne and Mason 1971; Goulet and Huber 1993). In the United Kingdom, three genera of Diapriinae were recorded in the Graham-Smith study on carrion. *Aneurhynchus* and *Psilus* were found in insect-open carrion, and *Trichopria* in buried carrion (Graham-Smith 1919; Payne and Mason 1971). In France, *Trichopria inermis* Kieffer, a gregarious parasitoid of *L. sericata*, was been observed (Blanchot 1995).

The last species identified in the laboratory was *N. vitripennis*, a gregarious ectoparasitoid of the pupae of several fly species of forensic importance, including blowflies, flesh flies, and houseflies (Whiting 1967; Rueda and Axtell 1985; Blanchot 1992). The attraction of females of this species toward the host can be caused, *in natura*, by decomposing meat (Laing 1937; Blanchot 1992; Frederickx et al. In Press). These wasps are regularly found on carcasses (Blanchot 1995; VanLaerhoven and Anderson 1999; Amendt et al. 2000; Grassberger and Frank 2004; Pohjoismaki et al. 2010) or in bird nests (Whiting 1967; King and Ellison 2005). This species has been recorded worldwide (Braack 1987). *N. vitripennis* was identified in Belgium in 1920 (Mitroiu 2001; Vago 2006), is a cosmopolitan species (Darling and Werren 1990; Yoder et al. 1994), and has been widely investigated in the subjects of genetic, ecological, evolutionary, and developmental research over the last 50 years (Darling and Werren 1990; Grassberger and Frank 2003; Steiner et al. 2006; Gadau et al. 2008). These wasps are commercially supplied and widely used as biological control agents of blowflies in Australia and in the United States (Mandeville et al. 1990; Morgan et al. 1991; Floate et al. 1999; Grassberger and Frank 2003). Only one pupa was parasitized by female *N. vitripennis. N. vitripennis* is not considered to be adapted for burrowing, and buried pupae are typically beyond the reach of parasitizing females (Altston 1920; Wylie 1958; Beard 1964; Whiting 1967; Vinson 1976; Voss et al. 2009). In 1950, a higher incidence of parasitism by this species in pupae located on or near the surface of a carcass, rather than on those buried in the soil was reported (Ullyett 1950). In the present study, no Diptera specimens were collected on the ground.

### Forensic interest

Forensic entomology is the application of the study of insect biology to criminal matters and is frequently used to estimate the time that has elapsed since death, or the post-mortem interval (Gennard 2007; Ricciuti 2007; Eberhardt and Elliot 2008). Forensic practitioners have previously postulated the use of parasitoids as a tool in criminal investigations, although the presence of parasitoids at crime scenes has largely been ignored due to their small size and the paucity of biological information available (Grassberger and Frank 2003; Amendt et al. 2007; Voss et al. 2009). Females of Hymenoptera parasitoids usually prefer to parasitize particular instars of their hosts (Reznik et al. 1992). In many cases, the most preferable stages are also the most suitable, creating optimal synchronization (Laing 1937; Vinson 1976; Reznik et al. 1992). *A. manducator* prefer larvae of *Calliphora vicina* that have already finished their feeding but have not yet left the food substrate (Reznik et al. 1992). *N. vitripennis* usually lay eggs in their host one day after the pupation, when the skin of the larva has separated from the inner pupal cuticle (Gunn 2006; Gaudry 2010). However, it has been reported that two dayold pupae were parasitized at a significantly lower rate than pupae exposed for four days (Kaufman et al. 2001).

Considering the abundance of *A. manducator, T. zealandicus*, and *Figites sp*., the use of these species as a reliable forensic indicator for estimating post-mortem interval is promising. In order to estimate post-mortem interval using these species, the calculated developmental time of the parasitoid simply has to be added to the time of development of the host, therefore providing an extended post-mortem interval timeframe in cases where traditional forensic indicators have completed their development (Grassberger and Frank 2003; Amendt et al. 2010). However, when considering the potential influence, especially of larval parasitoids, it is important to take into account that they can also create significant problems, as seen in the change of developmental times for *L. sericata* after attack of *A. manducator*, which results in a premature pupation (Holdaway and Evans 1930). This problem clearly illustrates the need for further research in this field.
